# Does infection of a mandibular fracture lead to the development of chronic pain syndrome? Assessment of patient treatment results based on functional indicators, standardized questionnaires and quality of life assessment

**DOI:** 10.22514/jofph.2025.018

**Published:** 2025-03-12

**Authors:** Lejli U. Valieva, Alexander S. Pankratov, Archil O. Mikiya

**Affiliations:** ^1^Department of Maxillofacial Surgery, I.M. Sechenov First Moscow State Medical University (Sechenov University), 119991 Moscow, Russia; ^2^Department of General and Surgical Dentistry, Russian Medical Academy of Continuous Professional Education, 125993 Moscow, Russia

**Keywords:** Fracture, Mandibular mobility, Pain syndrome, Inflammatory complication, Functional recovery

## Abstract

**Background**: Fractures of the mandible are among the most common injuries to the bones of the facial skeleton and are associated with a relatively high incidence of complications, particularly purulent-inflammatory conditions, especially when treatment is delayed. These complications and surgical interventions can damage the masticatory muscles, disrupt their physiological balance, impair mandibular movement and contribute to pain syndrome development. This study aimed to investigate the dynamics of pain severity, the restoration of stomatognathic apparatus function following purulent-inflammatory complications of mandibular fractures and their impact on patients’ quality of life. **Methods**: We assessed the data of 15 patients with mandibular fractures without fragment displacement but complicated by purulent-inflammatory processes. Surgical intervention was combined with intermaxillary immobilization for four weeks, followed by myogymnastic exercises during rehabilitation. Mandibular movement amplitude was measured in three planes and surveys were conducted. Pain syndrome was assessed using the Visual Analog Scale and McGill Pain Questionnaire, and their psycho-emotional status was evaluated using the Spielberger-Hanin Anxiety Scale and Beck Depression Inventory. Quality of life was measured using the Medical Outcomes Study-Short Form questionnaire (SF-36). Assessments were performed on the fourth postoperative day, immediately after splint removal,and at one, six and twelve months post-operation. Mandibular mobility was also measured seven and fourteen days post-splint removal. **Results**: The results were then compared with a group of healthy volunteers. Over one year of observation, we found that all functional and psychometric parameters of the patients remained significantly lower than those of the healthy volunteer group, and these deficits predisposed patients to muscle dysfunction and negatively impacted their quality of life. **Conclusions**: Therefore, continued research is essential to develop effective treatment and rehabilitation strategies for this patient population.

## 1. Introduction

Injuries to the bones of the facial skeleton represent a significant public health concern worldwide [1], with over 400,000 emergency department visits reported annually in the United States [2]. Mandibular fractures rank among the most prevalent, comprising 36% to 70% of these cases [3], and are frequently associated with complications, predominantly of an inflammatory nature, with reported incidence rates ranging from 4.5% to 30% [4, 5, 6, 7, 8]. Inflammatory complications of mandibular fractures are often localized at the attachment sites of the masticatory muscles [9]. These processes can follow a severe course, potentially spreading through the fascial spaces of the maxillofacial region and neck, and in rare cases result in fatal outcomes [10].

Traditionally, for immobilizing mandibular fragments in the presence of an inflammatory reaction at the injury site, dental splints and external fixation devices were used [4, 11]. With the introduction of internal fixation technology, this method has also been applied successfully under appropriate conditions, either concurrently with the incision and drainage of the purulent-inflammatory focus [4] or after the resolution of acute inflammation [12, 13]. Despite its clinical benefits, internal fixation has yet to achieve widespread adoption, primarily due to concerns among some practitioners regarding the placement of metal constructs “directly in pus”. Surgical interventions for incising and draining purulent-inflammatory foci often involve cutting the fibers of the masticatory muscles and detaching them from the bone. The secondary healing of purulent wounds typically leads to the formation of coarse scar tissue. Damage to the masticatory muscles disrupts their physiological balance with their counterparts and antagonistic muscles, adversely affecting the intricate interaction of facial and upper neck muscles. Consequently, such disruptions alter the mechanics of mandibular movement [14] and are consistently observed in these cases [15].

In 9% to 60% of cases, painful trigger zones develop in the projection of the masticatory muscles during the postoperative period following the incision of phlegmons [16]. Prolonged pain afferentation caused by trauma, the associated inflammatory reaction, and the required surgical intervention collectively impair the contractile ability of the masticatory muscles [17] and substantially limit mandibular mobility. Moreover, therapeutic measures involving intermaxillary immobilization further exacerbate this limitation. Extended immobilization of the mandibular head disrupts the production of synovial fluid within the temporomandibular joint (TMJ) [18, 19], contributing to joint disorders. The progression of these disorders is further influenced by peripheral and central sensitization, which arises from prolonged afferent nociception [20] (Fig. 1). This process underpins the development of chronic pain syndrome, which gradually becomes independent of peripheral triggers [21].

**Fig. 1.** The pathogenesis of the development of functional and painful disorders of the stomatognathic apparatus in trauma.

Purulent-inflammatory complications of mandibular fractures may predispose patients to the development of chronic pain syndrome. When combined with persistent functional disorders, this significantly impacts the quality of life. As societal expectations regarding treatment outcomes continue to rise, it becomes evident that addressing acute inflammatory phenomena, achieving bone fragment consolidation and restoring the anatomical configuration of the mandible is no longer sufficient to define clinical success. Instead, the final evaluation should encompass a range of indicators, including the functional activity of the stomatognathic apparatus, the presence or absence of pain and parameters of psychological well-being. Despite the importance of these factors, the current literature provides insufficient attention to the rehabilitation methods for this patient category or their effectiveness. Publications addressing this topic are notably scarce. Given this gap in knowledge, there is a need to evaluate the relationship between dental and psychological health indicators in patients with inflammatory complications of mandibular fractures, both in the short and long term following treatment completion.

Accordingly, the aim of this studywas to investigate the dynamics of pain severity, the restoration of the functional state of the stomatognathic apparatus following purulent-inflammatory complications of mandibular fractures, and their impact on patients’ quality of life.

Experimental hypothesis: Patients with purulent-inflammatory complications of mandibular fractures exhibit significant differences in functional and psychometric indicators in the long term (6 months post-injury) compared to healthy individuals.

Null Hypothesis: Patients with purulent-inflammatory complications of mandibular fractures do not exhibit significant differences in functional and psychometric indicators in the long term (6 months post-injury) compared to healthy individuals.

## 2. Materials and methods

The study was conducted on patients with mandibular fractures who presented to the clinic due to the development of purulent-inflammatory complications at the injury site. To minimize the influence of confounding factors on the results, patients with combined injuries to other anatomical regions, coexisting internal organ pathologies or a history of symptoms indicating pre-existing maxillofacial pathology before the injury were excluded. Given that the displacement of bone fragments exacerbates the severity of the injury and that performing bone osteosynthesis during a developed purulent-inflammatory process poses a significant risk, only cases of mandibular fractures without fragment displacement were included, and this approach eliminated the need for additional invasive surgical interventions.

The exclusion criteria for the study were as follows: (1) Cases with progression or recurrence of inflammatory phenomena within one year after surgery. (2) Patients who failed to attend scheduled examinations within the study timeline or did not comply with medical recommendations.

The comparison group comprised healthy volunteers who met the following criteria: no history of trauma or surgery in the maxillofacial region, no malocclusion or dental arch integrity issues and no symptoms of musculoskeletal, somatic or neurological disorders. Normal occlusion was preserved in all patients and healthy volunteers included in the study.

The study was conducted in the rehabilitation room of the Maxillofacial Surgery Department at Hospital No. 36 named after F.I. Inozemtsev, Moscow. The selection of patients who met the study criteria was carried out over the course of 2 years (2021-2022), followed by follow-up for a year. All patients provided informed consent for participation in the research, including the use of their data for scientific purposes and publication, in compliance with confidentiality requirements. All procedures adhered to the ethical standards of the institutional research committee and the principles of the 1964 Helsinki Declaration. The study was approved by the Local Ethics Committee of the First Moscow State Medical University named after I.M. Sechenov (Protocol No. 34–20, dated 09 December 2020).

The study included 15 patients with mandibular fractures complicated by purulent-inflammatory processes, all of whom reported delayed medical consultation following injury. Among them, 10 patients had unilateral fractures and 5 had bilateral fractures. The fracture lines were located in the lateral body and angle of the mandible. Upon admission, they underwent bimaxillary splinting with intermaxillary immobilization. Small bone fragments and teeth in the fracture line were removed as needed, and antiseptic treatment was administered. On the same day, purulent foci were incised and drained via an external approach. Antibacterial and anti-inflammatory therapies were initiated and continued until the acute inflammatory symptoms resolved. Symptomatic and vitamin therapies, supplemented with trace elements, were also provided. For all patients, a single surgical intervention was sufficient, with no progression or recurrence of purulent-inflammatory processes. The duration of intermaxillary fixation was four weeks, consistent with regulatory guidelines [6] and the expected timeline for primary bone callus formation in secondary bone healing [22]. Osteosynthesis was not performed in any of the studied patients.

After the removal of the fixation devices, the patients were prescribed a regimen of myogymnastic exercises to increase muscle stretch amplitude and gradually enhance muscle tone and strength. The exercises were performed systematically, 4–5 times daily, and included movements in the vertical, transverse and sagittal planes, with and without resistance (Fig. 2).

**Fig. 2.** Examples of myogymnastical exercises used. (A) Opening and closing of the mouth with the tongue pressed against the palate. (B) Lateral movements of the mandible with the mouth closed. (C) Protrusion of the mandible with the mouth closed. (D) Mouth slightly open. Fingers of both hands are placed on the chin and gently pull the mandible downward. The masticatory muscles are tensed to prevent lateral displacement of the mandible, which is monitored using a mirror. (E) Forced increase in the amplitude of mouth opening using fingers. Index fingers are placed on the occlusal surfaces of the lower molars, while the thumbs are on the upper molars. (F) Forced increase in the amplitude of lateral movements of the mandible. (G) Forced increase in the amplitude of anterior movements of the mandible. Fingers of both hands grasp the chin from both sides. (H) Opening of the mouth against moderate resistance from the hand. (I) Lateral movements of the mandible against moderate resistance from the hand. (J) Protrusion of the mandible against moderate resistance from the hand.

Four days after surgical intervention (study point T0), patients underwent clinical and radiological evaluations along with a comprehensive survey. The following assessment tools were used:

Visual Analog Scale (VAS): Patients marked pain intensity on a 10 cm line, where 0 indicated no pain and 10 represented the worst pain imaginable. Pain intensity was categorized as very mild (0–1 points), mild (2–4 points), moderate (4–6 points), very severe (6–8 points), and unbearable (8–10 points).

The McGill Pain Questionnaire [23] consists of 78 pain-descriptive terms grouped into 20 subclasses. Within each subclass, descriptors are organized in ascending order of pain intensity. The questionnaire generates two primary indicators: the Pain Rating Index (PRI), which quantifies the severity of pain, and the Number of Words Chosen Index (NWC), which reflects the range of descriptive terms selected by the patient.

Psycho-emotional status was assessed using the Beck Depression Inventory [24] and the Spielberger-Hanin Anxiety Scale [25], and quality of life was evaluated using the SF-36 questionnaire [26].

Subsequent assessments were conducted at specific study points: the day of splint removal (T1), one month later (T2), six months later (T3) and twelve months later (T4) following the start of rehabilitation. Concurrently, starting from T1, the degree of mandibular mobility was measured at 7 and 14 days after splint removal and at study points T2, T3 and T4. Measurements were performed using a medical caliper, and the results are expressed in millimeters.

Maximum mouth opening was determined by measuring the distance between the incisal edges of the upper and lower central incisors. Protrusion was evaluated by instructing the patients to move their mandibles forward, with the horizontal overlap between the central incisors of the upper and lower jaws being measured. Laterotrusion was assessed differently depending on the type of fracture.

For unilateral fractures, lateral mandibular movements toward the injured and uninjured sides were measured. For bilateral fractures, the lateral movements on both injured sides (right, referred to as side 1, and left, referred to as side 2) were assessed. The horizontal distance between the central inter-incisal lines of the upper and lower jaws was recorded following the lateral movement of the mandible in the corresponding direction. For cases with midline deviation of the central incisors before injury, appropriate adjustments were applied during measurements.

The study points T0, T1, T2, T3 and T4, during which patients underwent comprehensive evaluations according to the described methods, were designated as the main assessment points. Measurements conducted at 7 and 14 days after splint removal, focusing exclusively on the amplitude of mandibular movements, were regarded as additional (secondary) assessments. The data obtained from these assessments were compared with those of a control group, which consisted of 10 healthy volunteers aged 18 to 52 years. Each individual in the control group underwent a single evaluation using the same methodologies. The distribution of patients across comparison groups is presented in Table 1.

**Table 1.** Characteristics of patients in the comparison groups.

Groups		n		
The studied patients				
	Age (yr)	18–30	31–40	41–52
	Men	3	5	3
	Women	2	2	0
	Total	5	7	3
Healthy volunteers				
	Age (yr)	18–30	31–40	41–52
	Men	2	2	1
	Women	3	1	1
	Total	5	3	2

Statistical analysis was performed using IBM SPSS (Version 27.0, Armonk, NY, USA) Statistics for Windows, software on the MS Windows (Version 22H2, Redmond, WA, USA) platform. Mean values and their standard deviations were calculated using standard statistical methods. Comparisons between patients with purulent-inflammatory complications of mandibular fractures and healthy volunteers were conducted using the Mann-Whitney U test for independent samples. Statistical significance was defined as *p *≤ 0.05.

## 3. Results

### 3.1 Pain syndrome

The dynamics of pain syndrome intensity in patients with purulent-inflammatory complications of mandibular fractures, as measured using VAS, are presented in Table 2. During the jaw immobilization period, all patients reported increased pain during eating, particularly while engaging in active sucking movements to consume liquid food through a straw (jaw diet). Pain was further aggravated by talking, minimal emotional stress, jaw clenching and prolonged standing. Patients also frequently reported rapid fatigue when performing these activities. Half of the patients experienced worsened pain at night, often struggling to find a comfortable head position without exacerbating facial pain. Although analgesics provided partial relief, residual pain persisted in the masticatory muscles, TMJ and oral cavity due to the presence of splinting devices. Some patients described episodes of sudden, intense, constricting pain on the affected side, resembling acute painful trismus, which occasionally resolved spontaneously but more often required analgesics or massage for relief. Additionally, some reported involuntary muscle contractions resembling “cramps”, primarily on the injured side.

**Table 2.** Dynamics of changes in the multidimensional pain assessment index according to the McGill Pain questionnaire and the intensity of pain syndrome in the masticatory muscles according to VAS in patients with purulent-inflammatory complications of mandibular fractures.

Indicator	Patients under Study					Healthy Volunteers
	Т0	Т1	Т2	Т3	Т4	
PRI Sensory Class	10.27 ± 3.31*	6.80 ± 1.78*	5.07 ± 1.22*	3.47 ± 1.30*	4.13 ± 1.68*	0
NWC Sensory Class	5.60 ± 1.24*	4.40 ± 0.83*	3.27 ± 0.59*	2.53 ± 0.64*	2.73 ± 0.70*	0
PRI Affective Class	5.47 ± 2.07*	4.60 ± 1.35*	3.93 ± 0.79*	3.67 ± 0.98*	3.40 ± 0.91*	0
NWC Affective Class	3.67 ± 0.62*	3.60 ± 0.68*	3.33 ± 0.62*	3.20 ± 0.60*	3.13 ± 0.52*	0
VAS	7.51 ± 0.54*	5.92 ± 0.46*	4.65 ± 0.60*	3.64 ± 0.63*	3.82 ± 0.64*	0

*p < 0.05, significance of differences compared to healthy volunteer group. PRI: Pain Rating Index; NWC: Numberof Words Chosen; VAS: Visual Analog Scale.

At T1 (the day of splint removal), VAS scores indicated a 21% reduction in pain intensity compared to baseline (T0). However, clinical examinations revealed dense consistency, tension and tenderness in the masticatory muscles on both sides, irrespective of the type of fracture. Trigger points were detected in some patients, where irritation elicited paroxysmal, constricting pain. One month after splint removal (T2), pain intensity had decreased further by 38% relative to T0, and by six months (T3), the reduction reached 51.5%. Despite this improvement, most patients reported rapid fatigue of the masticatory muscles and feelings of tiredness during chewing and talking. Morning pain was commonly described as “pulling”, requiring myogymnastic exercises and massages to alleviate muscletension. After one year of rehabilitation treatment (T4), no further reduction in pain intensity was observed compared to T3. Instead, there was a slight increase in average pain intensity by 4.95%, and trigger points in the masticatory muscles remained palpable.

The dynamics of changes in the McGill Pain Questionnaire indices are summarized in Table 2. After the immobilization period (T1), the PRI and NWC for the sensory class showed reductions of 33.79% and 21.43%, respectively, compared to baseline (T0). In contrast, the reduction in PRI for the affective class was less pronounced at 15.91%, while the NWC for the affective class remained approximately unchanged from T0. At this stage, the sensory class of pain predominated. One month later (T2), further reductions were observed in the sensory class indices, with PRI and NWC decreasing by 25.44% and 25.68%, respectively, relative to T1. However, the reductions in PRI and NWC for the affective class remained less significant, at 14.57% and 7.5%, respectively. At six months (T3), the sensory class continued to decline, with PRI and NWC decreasing by 31.59% and 22.63%, respectively, compared to T2. The affective class showed less pronounced reductions during this period, with PRI and NWC decreasing by 6.62% and 3.9%, respectively. By twelve months (T4), a reversal was observed in the sensory class, with PRI and NWC increasing by 19.02% and 7.91%, respectively, relative to T3. Meanwhile, the affective class showed slight reductions in PRI and NWC, at 7.36% and 2.19%, respectively. By the end of the study, when compared to baseline (T0), the sensory class demonstrated significant overall reductions in PRI and NWC by 59.79% and 51.25%, and in the affective class, the reductions were 37.84% and 14.71%, respectively.

### 3.2 Psycho-emotional status

The changes in reactive and personal anxiety levels among the studied patients are summarized in Table 3. On the fourth day after surgery (T0), reactive anxiety levels were significantly elevated, nearly twice as high as those observed in healthy volunteers. Patients expressed concerns related to their appearance, the prolonged use of splinting devices, adherence to a restrictive jaw diet, the extended rehabilitation process and the potential for delayed complications. By the end of the jaw immobilization period (T1), the average reactive anxiety level had decreased by 10.1% compared to T0, although it remained high, at 48.07 ± 10.13 points. One month later (T2), the average level had decreased by 15.2%, and six months later (T3), by 21.3%. After twelve months of rehabilitation (T4), reactive anxiety had decreased by 28.2% relative to baseline, with a final value of 38.4 ± 8.42 points, corresponding to a moderate level of reactive anxiety.

**Table 3.** Dynamics of changes in the psycho-emotional state in studied patients with purulent-inflammatory complications of mandibular fractures, according to the Spielberger scale modified by Khanin and to the Beck Depression Inventory (points).

Indicator	Patients under Study					Healthy Volunteers
	Т0	Т1	Т2	Т3	Т4	
Reactive anxiety levels	53.47 ± 11.08*	48.07 ± 10.13*	45.33 ± 8.79*	42.07 ± 8.64*	38.40 ± 8.42*	24.40 ± 1.20
Personal anxiety levels	28.94 ± 5.56	33.87 ± 4.63*	38.07 ± 5.47*	39.60 ± 6.34*	47.40 ± 9.41*	25.80 ± 0.80
Depression levels	20.10 ± 4.71*	23.33 ± 4.20*	19.80 ± 3.09*	18.67 ± 2.69*	20.40 ± 4.73*	4.70 ± 1.70

*p < 0.05, significance of differences compared to healthy volunteer group.

At baseline (T0), personal anxiety levels were low, with an average of 28.94 ± 5.56 points. However, a progressive worsening in personal anxiety was observed over the course of the study. By the time of splint removal (T1), personal anxiety had risen to 33.87 ± 4.63 points, corresponding to a moderate level. One month later (T2), average personal anxiety values were 31.6% higher than baseline. Six months into the study (T3), these values had increased by 36.8%, and after twelve months (T4), they were 63.8% higher, reaching 47.4 ± 9.41 points, which corresponds to a high level of personal anxiety.

According to the Beck Depression Inventory (Table 3), the initial depression score at T0 was 20.1 ± 4.71 points, corresponding to the lower boundary of the severe depression category. By the end of the jaw immobilization period (T1), the average score had increased by 16.1% compared to baseline, reaching 23.33 ± 4.2 points, which remained within the severe depression range. One month after splint removal and the initiation of myogymnastic exercises (T2), a positive trend was observed, with depression scores decreasing by 15.1% relative to T1, returning closer to baseline levels. By six months (T3), depression scores further decreased to 18.67 ± 2.69 points, corresponding to a moderate level and reflecting a 5.7% reduction compared to T2. However, at twelve months (T4), a deterioration in depressive symptoms was noted, with an 9.3% increase in scores relative to T3, ultimately exceeding the baseline value by 1.5%.

### 3.3 Mandibular mobility

The values for mandibular movement amplitude in the vertical, transverse and sagittal planes at various study periods are presented in Table 4. The results were compared not only to the indicators of the healthy volunteer group but also to the lower limits of normal mandibular mobility, defined in the literature as 4.5 cm for mouth opening and 0.7 cm for forward and lateral mandibular movements [27, 28].

**Table 4.** Dynamics of mandibular motion amplitude in three planes in patients with purulent-inflammatory complications of mandibular fractures during rehabilitation.

Mandibular Movements (cm)	Observation Period						Healthy Volunteers
	T1	7 d	14 d	T2	T3	T4	
Maximum Mouth Opening	0.52 ± 0.25^#^	1.19 ± 0.45^#^	1.68 ± 0.54^#^	2.28 ± 0.27^#^	2.84 ± 0.31^#^	3.13 ± 0.29^#^	5.02 ± 0.10
Protrusion	0.10 ± 0.09^#^	0.19 ± 0.08^#^	0.26 ± 0.07^#^	0.31 ± 0.09^#^	0.41 ± 0.09^#^	0.47 ± 0.07^#^	1.02 ± 0.08
Laterotrusion to Injured Side in Unilateral Fractures	0.14 ± 0.05^#^	0.33 ± 0.05^#^	0.41 ± 0.06^#^	0.46 ± 0.05^#^	0.60 ± 0.05^#^	0.71 ± 0.07^#^	1.10 ± 0.08
Laterotrusion to Uninjured Side in Unilateral Fractures	0.16 ± 0.05^#^	0.31 ± 0.09^#^	0.39 ± 0.09^#^	0.41 ± 0.07^#^	0.54 ± 0.07^#^	0.61 ± 0.06^#^	1.10 ± 0.08
Laterotrusion to Injured Side-1 (Right) in Bilateral Fractures	0.15 ± 0.05^#^	0.30 ± 0.1^#^	0.43 ± 0.11^#^	0.45 ± 0.08^#^	0.62 ± 0.11^#^	0.70 ± 0.07^#^	1.10 ± 0.08
Laterotrusion to Injured Side-2 (Left) in Bilateral Fractures	0.20 ± 0.1^#^	0.34 ± 0.09^#^	0.44 ± 0.11^#^	0.46 ± 0.09^#^	0.54 ± 0.11^#^	0.58 ± 0.08^#^	1.10 ± 0.08

*^#^p < 0.05, significance of differences compared to healthy volunteer group.*

Throughout the study, mandibular mobility progressively improved across all three planes. However, by the end of the study period (T4), the mouth-opening amplitude remained 30.4% below the normal limit and 37.6% lower than the values observed in the healthy volunteer group. A similar pattern was observed for protrusion. One year after splint removal, mandibular forward movement was 32.9% below the normal limit and 53.92% lower than the healthy volunteer group average.

In unilateral mandibular fractures, lateral mandibular movement towards the injured side reached the lower normal limit by the end of the study period (T4) but remained 35.5% below the corresponding values in the healthy volunteer group. Conversely, lateral movement towards the uninjured side was 12.86% below the normal lower limit and 44.55% below the healthy volunteer group average.

In patients with bilateral fractures, lateral mandibular movement towards injured side-1 (right) achieved the lower normal limit by T4 but was still 36.36% below the values observed in the healthy volunteer group. Lateral movement towards injured side-2 (left) failed to reach the normal limit throughout the study period, remaining 17.14% below the normal threshold and 47.27% below the healthy volunteer group average. The observed differences in lateral movement between the injured sides are likely attributable to the predominance of fractures causing purulent-inflammatory complications on the right side in the studied patients.

### 3.4 Quality of life

The dynamics of qualityof life changes in the studied patients, as assessed by the SF-36 questionnaire, are shown in Fig. 3. At baseline, both physical and mental health components were significantly reduced, and could be attributed to factors such as pain, dietary restrictions, communication limitations, prolonged disability and the disruption of normal life rhythms.

**Fig. 3.** Dynamics of changes in quality of life according to the physical and psychological health component scales of the SF-36 questionnaire in studied patients with suppurative-inflammatory complications ofmandibular fractures. *p < 0.05 significance of differences compared to the group of healthy volunteers. PF: physical functioning; RP: role-physical functioning; BP: bodily pain; GH: general health; VT: vitality; SF: social functioning; RE: role-emotional functioning; MH: mental health.

By the end of the jaw immobilization period (T1), no significant changes were observed in the physical health component compared to baseline, with a value of 34.09 ± 2.98 points. However, the mental health component declined by 38.6% to 21.40 ± 7.47 points. Compared to the average indicators (58.68 and 57.25 points) of the healthy volunteer group, these values were 41.91% and 62.62% lower, respectively.

One month after splint removal (T2), slight improvements were observed. The physical health component increased by 1.44%, reaching 34.58 ± 3.12 points, while the mental health component increased by 37.06%, reaching 29.33 ± 6.24 points, compared to T1. Despite these improvements, the physical and mental health components remained 41.07% and 48.77% lower than the healthy volunteer group averages.

Six months after the start of rehabilitation treatment (T3), further positive trends were observed. The physical health component increased by 5.67% to 36.54 ± 3.17 points, and the mental health component increased by 18.04% to 34.62 ± 6.46 points, compared to T2. However, these values were still 37.73% and 39.53% lower, respectively, than the healthy volunteer group averages.

After twelve months of rehabilitation (T4), the physical health component increased by 7.01% to 39.10 ± 3.35 points, and the mental health component increased by 7.25% to 37.13 ± 6.79 points, compared to T3. While showing significant improvement, these values remained 33.37% and 35.14% lower, respectively, than the average indicators of the healthy volunteer group.

## 4. Discussion

The results of this study confirm the experimental hypothesis across all evaluated indicators. Even one year after the successful treatment of inflammatory complications of mandibular fractures, including the consolidation of bone fragments and the complete resolution of inflammatory phenomena, full restoration of stomatognathic system function was not observed, despite adherence to regular myogymnastic exercises.

Although the intensity of pain syndrome significantly decreased during the rehabilitation process, it did not fully resolve. Notably, six months after the removal of fixation devices, the trend of further reductions in VAS scores and PRI and NWC scores for the sensory class of the McGill Pain Questionnaire decreased. Similarly, while PRI and NWC scores for the affective class also decreased, these parameters continued to reflect a substantial contribution from peripheral and psycho-emotional pain components by the end of the study. These findings indicate the development of chronic pain syndrome, which may act as a trigger for additional pathological conditions, particularly those affecting the TMJ [18, 19]. Persistent pain interferes with the effective performance of myogymnastic exercises, and in some cases, discourages patients from continuing these exercises altogether. This further exacerbates the impairment of functional parameters.

These findings align with the observations of S.Y. Serpinov [16], who reported the development of trigger points in the masticatory muscles following surgery. The concept of myofascial trigger points, first introduced by J. Travell, defines them as “hyperirritable spots usually located within a taut band of skeletal muscle or in the muscle fascia, painful on compression and capable of producing characteristic referred pain, tenderness and autonomic phenomena” [29]. In our study, approximately half of the patients exhibited trigger points in the masticatory muscles throughout the observation period, with their prevalence increasing in some cases over time. These trigger points appear to serve as sources of pathological impulses transmitted to higher centers of the central nervous system when the masticatory muscle is tensed or stretched during normal functioning. Their persistence may contribute to the perpetuation of chronic pain and hinder the restoration of normal muscle function, further emphasizing the need for targeted interventions to address these issues.

The presence of chronic pain syndrome creates conditions conducive to the development of neurological symptoms [20, 21]. As emphasized by M. Oksa *et al*. [4], pain management is one of the primary objectives in treating patients with inflammatory complications of mandibular fractures, alongside occlusal restoration and the elimination of inflammation. However, an adequate and comprehensive program for achieving effective pain management has yet to be developed. The results of this study indicate that routine analgesic administration, as part of symptomatic therapy, is insufficient to address this issue.

The studied patients exhibited significant restrictions in mandibular mobility throughout the rehabilitation period. Only unilateral lateral movements towards the injured side in cases of unilateral fractures and towards the less injured side-1 (right) in bilateral fractures reached the lower limits of normal after one year. Given that the more severe injuries causing purulent-inflammatory processes were predominantly localized on the right side, this phenomenon is likely attributable to hypertonicity in the uninjured (in unilateral fractures) or less injured (in bilateral fractures) masticatory muscles on the opposite side. However, even in these cases, the observed mobility indicators remained substantially lower than those of the healthy volunteer group.

The primary trauma, the subsequent development of a purulent-inflammatory focus, and the surgical intervention for its drainage collectively exert a direct and significant damaging effect on the masticatory muscles. This cascade of events provokes a specific response in muscle tissue, characterized by increased reflex excitability, tension and eventual contraction [30], inevitably leading to a pronounced muscle imbalance, as previously noted, which manifests as impaired mandibular mobility [14]. Chronic pain syndrome, driven by substantial afferent input from various receptors in the trauma area, including those in the oral mucosa, periodontal tissues, alveolar nerves and masticatory and facial muscles, persists for an extended period. This pain intensifies under functional load, further reinforcing the existing muscle imbalance [31, 32]. Consequently, patients exhibit slower mandibular movements with reduced amplitude. As highlighted by K.W. Dos Santos* et al*. [33], the extent of soft tissue damage is directly associated with more pronounced protective reactions, such as restricted mandibular movements. Electromyographic studies conducted by S. Bither *et al*. [34], demonstrated that the muscle activity of injured masticatory muscles in patients with mandibular angle fractures remains significantly lower than that of healthy individuals of similar age and sex, even six months after trauma. The findings of this study suggest that in patients with inflammatory complications of mandibular fractures, this muscle imbalance may persist for an even longer period, potentially becoming chronic. To confirm this hypothesis and gain a deeper understanding of the underlying mechanisms, future studies should include electromyographic assessments conducted over extended post-trauma intervals.

Prolonged immobilization of the masticatory muscles in a non-physiological state during intermaxillary immobilization likely contributes to the progression of muscle imbalance and the formation of scar tissue. However, there are conflicting data on the recovery dynamics of functional parameters using different treatment methods in existing literature. For example, M. Schneider *et al*. [35], and S. Nogami *et al*. [36], reported that patients treated with open reduction and rigid fixation of bone fragments experienced less discomfort, better mandibular mobility during mouth opening, and more favorable regeneration compared to those undergoing closed reduction methods. In contrast, A.R. da Silva *et al*. [37], found greater restrictions in all mandibular movements, particularly maximum mouth opening, in patients treated with open reduction, attributing these limitations to surgical trauma. These contradictory findings preclude definitive conclusions regarding patients with inflammatory complications of mandibular fractures, for whom open reduction with rigid fixation involves a more invasive surgical intervention. Further research is warranted to address this issue comprehensively.

The presence of chronic pain syndrome and impaired stomatognathic system function significantly impacted the psycho-emotional state and quality of life of the studied patients. Reactive anxiety levels only decreased to a moderate level after one year of observation, while personal anxiety increased to a high level. Although depressive symptoms fluctuated over time, they ultimately returned to baseline levels after one year, corresponding to a severe depression category on the Beck Scale. These findings align with existing literature [38, 39, 40], though the changes observed in this study were more pronounced. For example, P. Sen *et al*. [38], reported that approximately 30% of patients with facial skeletal fractures experienced anxiety or depression one year post-trauma, whereas this study observed these issues in all patients, with no clear trend toward improvement in psychological status over time.

The identified morphological-functional and psycho-emotional disturbances directly influenced the patients’ quality of life. By the end of the study, quality-of-life indicators in the physical health component were 33.37% lower, and those in the mental health component were 35.14% lower than the healthy volunteer group. These findings indicate that the goal of achieving complete medical and social rehabilitation for these patients has not been fully realized.

## 5. Conclusions

In patients with mandibular fractures without fragment displacement complicated by purulent-inflammatory processes, full restoration of mandibular mobility does not occur even one year after treatment with intermaxillary immobilization. Despite the complete resolution of inflammatory phenomena, consolidation of bone fragments, regular myogymnastic exercises and symptomatic therapy, chronic pain syndrome persists. This condition adversely affects the psycho-emotional state and quality of life of these patients. Given the limited sample size of this study, further research is necessary to confirm these findings with more robust statistical data.

Clinical observations suggest the development of persistent muscle dysfunction in this patient population. To validate this hypothesis, electromyographic measurements of the functional parameters of the masticatory muscles at extended intervals post-trauma are required.

In this paper, the authors employed a strictly defined patient model to ensure focused observations. In this regard, a number of restrictive factors were applied: the absence of displacement of bone fragments and their operative fixation, the absence of signs of combined trauma and diseases of internal organs, the absence of cases of recurrence of the inflammatory process, the lack of data on the pathology of the maxillofacial region before the injury. In addition, a mandatory requirement was the patient’s commitment to rehabilitation treatment throughout the entire follow-up period. Although the findings presented highlight the need to improve rehabilitation methods for patients with such complications, further studies are needed to analyze a broader range of clinical scenarios and fully understand the long-term consequences of purulent-inflammatory complications of mandibular fractures. The next step may be to conduct randomized clinical trials of the use of various rehabilitation programs for the dynamics of recovery of functional and psychometric parameters in this category of patients. Another important area of scientific research is the study of the effect that prolonged intermaxillary immobilization has on this process, compared with early functional stress after functionally stable osteosynthesis performed despite the existing risk.
